# Growth Phase, Oxygen, Temperature, and Starvation Affect the Development of Viable but Non-culturable State of *Vibrio cholerae*

**DOI:** 10.3389/fmicb.2016.00404

**Published:** 2016-03-30

**Authors:** Bin Wu, Weili Liang, Biao Kan

**Affiliations:** ^1^State Key Laboratory for Infectious Disease Prevention and Control, National Institute for Communicable Disease Control and Prevention – Chinese Center for Disease Control and PreventionBeijing, China; ^2^Jiangsu Province Center for Disease Control and PreventionNanjing, China; ^3^Collaborative Innovation Center for Diagnosis and Treatment of Infectious DiseasesHangzhou, China

**Keywords:** viable but non-culturable state, *Vibrio cholerae*, culturability, starvation, oxygen limitation, growth phases, temperature

## Abstract

*Vibrio cholerae* can enter into a viable but non-culturable (VBNC) state in order to survive in unfavorable environments. In this study, we studied the roles of five physicochemical and microbiological factors or states, namely, different strains, growth phases, oxygen, temperature, and starvation, on the development of VBNC of *V. cholerae* in artificial sea water (ASW). Different strains of the organism, the growth phase, and oxygen levels affected the progress of VBNC development. It was found that the VBNC state was induced faster in *V. cholerae* serogroup O1 classical biotype strain O395 than in O1 El Tor biotype strains C6706 and N16961. When cells in different growth phases were used for VBNC induction, stationary-phase cells lost their culturability more quickly than exponential-phase cells, while induction of a totally non-culturable state took longer to achieve for stationary-phase cells in all three strains, suggesting that heterogeneity of cells should be considered. Aeration strongly accelerated the loss of culturability. During the development of the VBNC state, the culturable cell count under aeration conditions was almost 10^6^-fold lower than under oxygen-limited conditions for all three strains. The other two factors, temperature and nutrients-rich environment, may prevent the induction of VBNC cells. At 22 or 37°C in ASW, most of the cells rapidly died and the culturable cell count reduced from about 10^8^ to 10^6^–10^5^ CFU/mL. The total cell counts showed that cells that lost viability were decomposed, and the viable cell counts were the same as culturable cell counts, indicating that the cells did not reach the VBNC state. VBNC state development was blocked when ASW was supplied with Luria-Bertani broth (LB), but it was not affected in ASW with M9, suggesting that specific nutrients in LB may prevent the development of VBNC state. These results revealed that the five factors evaluated in this study had different roles during the progress of VBNC induction. Changing a single factor could influence and even block the development of the VBNC state. These findings provide new insight to help design further studies to better understand the mechanisms which trigger the development and regulation of the VBNC state.

## Introduction

The viable but non-culturable (VBNC) state is defined as a state where bacteria are metabolically active but lack the ability to reproduce on routine culture media (Oliver, 2005). Many bacteria, including a variety of important human pathogens, can enter into the VBNC state in the presence of unfavorable environmental conditions (Ramamurthy et al., 2014). It has been suggested to be a survival strategy by which bacteria are able to withstand such conditions (Pinto et al., 2015). While the ability to enter VBNC is advantageous for the survival of bacteria, it poses a risk to public health (Li et al., 2014). For instance, studies have found that *Vibrio cholerae* cells in the VBNC state can be resuscitated by introduction in a rabbit ileal loop and in the intestines of human volunteers (Colwell et al., 1985; Kaper et al., 1995). For other pathogens such as *Escherichia coli*, *V. vulnificus*, and *V. parahaemolyticus*, the resuscitation can be achieved by a mere increase in the temperature (Oliver and Bockian, 1995; Wong et al., 2004; Pinto et al., 2011).

Since the first report of VBNC in Xu et al. (1982), many studies have been devoted to explain the VBNC state (Oliver, 2005). However, very little is known about the transition to the VBNC state, especially the genetic mechanism behind it (Trevors, 2011). Instead, various conditions that can induce the VBNC state have been reported (Oliver, 2005, 2010; Pinto et al., 2015). Indeed, evaluation of the culture conditions that affect the development of the VBNC state may point to the underlying genetic regulation. However, the factors reported to induce non-cultivability appear conflicting among different studies (Pinto et al., 2015). One possible reason for this may be that the conditions studied were always complex, with several possible factors in combination. Factors such as starvation, temperature, oxygen level, sunlight, and salt concentration may all influence the VBNC process and lead to the instability. Although, many factors together contribute to inducing the VBNC state, the role of each factor may differ during the process and should be estimated (Trevors, 2011; Pinto et al., 2015). Identifying the specific factors that speed up or impede the development of the VBNC state may shed insights into the mechanisms underlying the triggering and development of the VBNC state.

Not much is known about the role of a single factor during VBNC development. Nevertheless, low temperature (5°C) alone could induce the VBNC state in *V. vulnificus* with or without starvation (Oliver et al., 1991). However, *Aeromonas hydrophila* and *Listeria monocytogenes* could be induced to the VBNC state at room temperature as well as at 5°C (Besnard et al., 2002; Maalej et al., 2004). Furthermore, the same set of conditions resulted in VBNC progressing at different pace even within the same bacterial species (Hoff, 1989; Byrd and Colwell, 1990; Besnard et al., 2002). These findings suggested that different factors may induce the VBNC state of different species in different ways (Besnard et al., 2002). Comparative studies on the effect of diverse factors inducing VBNC in different bacterial species are necessary to better understand this phenomenon.

This study aimed to determine the influences of some common environmental and microbiological factors on the development of VBNC in *V. cholerae*. In a laboratory setting, VBNC is usually induced in *V. cholerae* by incubating the culture in artificial sea water (ASW) at 4°C (Asakura et al., 2007). Some microbiological (including different biotype strains or growth phases) and physicochemical factors (including oxygen level, temperature, and nutrients) may play a part during the induction. To illustrate their influence, we studied and compared the effect of these factors on the induction of the VBNC state in *V. cholerae*.

## Materials and Methods

### Bacterial Strains and Preparation of VBNC Induction

*Vibrio cholerae* O1 serogroup classical biotype strain O395 and two O1 El Tor biotypes strains, namely, C6706 and N16961 (preserved in our laboratory) were used in this study. These strains were initially stored in 20% (v/v) glycerol at -80°C, then cultured on nutrient agar.

Three single colonies of each strain were picked and suspended in Luria-Bertani broth (LB; Oxoid, UK) and cultured with shaking (200 rpm) at 37°C overnight. The cultures were then diluted in fresh LB broth (1:50, v/v), incubated with shaking at 200 rpm at 37°C and grown to mid-exponential-phase and stationary-phase. The cultures were then washed twice with ASW and diluted to OD_600_ = 1.0 (approximately 1 × 10^9^ CFU/mL). Finally, the cultures were inoculated at a final concentration of 1 × 10^8^ CFU/mL into different media for use in subsequent experiments.

### Culture Media for VBNC Induction

Three culture media (ASW, ASW-LB, and ASW-M9) were used in this study. ASW was made of 40 g/L sea salt (Sigma, USA) and sterilized by passing through a 0.22 μm membrane filter (Millipore, USA). ASW-LB was prepared by mixing ASW with 10× of LB (9:1, v/v) to make a final concentration of 1% tryptone and 0.5% yeast extract. ASW-M9 was prepared by mixing ASW with 10× of M9 (9:1, v/v) to obtain a final concentration of 0.4% glucose as the carbon source and 0.1% NH_4_Cl as the nitrogen source.

### Conditions for the Induction of VBNC

**Table 1** presents the different culture conditions studied to determine their effect on VBNC induction. Each factor was studied individually and a control was set up for each strain (exponential-phase cells were cultured in ASW at 4°C with oxygen) for comparison. Oxygen limitation was achieved by filling 2 mL vials to the brim with the cultures and closing the lid to exclude air. At every time point, a new vial of each strain was used for the enumeration, and then disposed to prevent aeration. For presence of oxygen, each strain was incubated in a T75 flask with a 0.2 μm vent cap to facilitate aeration.

**Table 1:** Conditions used to study the factors in this study.

Conditions	Factors			
	Exponential	Oxygen	4°C	ASW
Control	+	+	+	+
Cell age	-	+	+	+
Oxygen	+	-	+	+
Temperature	+	+	-	+
Starvation	+	+	+	-

*“+” means the factor was the same as the control condition. “-” means the factor was changed.*

### Enumeration of Cultivable Cells

Culturable cells were enumerated by plating on tryptic soy agar (TSA; Oxoid, USA) supplemented with 0.1% sodium pyruvate (SP; Amresco, USA). The first time point of non-culturable cells could be formed from a 1 mL microcosm was considered as the time taken to lose culturability in 100% of the cell population (LCT100). The time taken for more than 50% and more than 90% of cells to lose their cultivability were termed LCT50 and LCT90, respectively (**Supplementary Data Sheet 1**).

### Enumeration of Viable Cells

As described in our previous study (Wu et al., 2015), viable cells were enumerated using propidium monoazide (PMA, Biotium, USA) combined with quantitative PCR (PMA-qPCR) by counting DNA copies of live cells. Briefly, 200 μL aliquots of cells cultured in each condition were treated with 20 μM of PMA for 20 min in the dark, then exposed to light on ice for 15 min using a 650W double-ended halogen lamp. Then, DNA was isolated from the 200 μL aliquots using a TIANamp Bacteria DNA Kit (Tiangen, China) according to the manufacturer’s instructions. qPCR was performed on a CFX96 Real-Time System (Bio-Rad, USA) using SYBR Premix Ex Taq (TaKaRa, Japan) with primers (forward primer, 5′-TAACATAATAAGGAAGAAGTGGAT-3′; and reverse primer, 5′-ACAGTCAGAAGCAGAGAA-3′) targeting the single copy gene VC1376 of *V. cholerae* O1. A plasmid DNA standard (forward primer, 5′- TTACCACTGACCTGAAGCGT-3′; and reverse primer, 5′-CAGGCGCACTTTATCCGAAA-3′) was constructed by introducing the DNA fragment into the pMD19 T-Vector (TaKaRa) according to the manufacturer’s instructions. The DNA copies recorded in this study were computed from the calibration curves generated from the dilutions of the plasmid DNA standard ranging from 1 × 10^7^ to 1 × 10^1^ DNA copies/μL by the Bio-Rad CFX manager 3.0 (**Supplementary Data Sheet 1**).

### Live/Dead Staining

Live/dead staining was performed as described before (Almagro-Moreno et al., 2015). We centrifuged 1 mL aliquots from each sample at 10,000 rpm for 1 min, and the pellet was suspended in 1 mL phosphate-buffered saline. The cells were then stained with a 3 μL mixture (1:1) of SYTO9 and propidium iodide per 1 mL of the suspension for nucleic acid staining (Molecular Probes, Eugene, OR, USA). The final concentrations of SYTO9 and propidium iodide were 5 and 30 μM, respectively. After incubation in the dark for 15 min at 25°C, the stained cells were mounted on a glass slide and low-fluorescence immersion oil was added on the cover slide. The cells were then examined with a Nikon ECLIPES 80i microscope. The images were captured with NIS-Elements F3.2 microscopy software (Nikon). According to the introduction of the manufacturer, green cells represent viable cells, being stained only by SYTO9.

### Statistical Analysis

The figures were drawn using GraphPad Prism software from three replicate values from three different colonies of each strain. Each replicate value represented the mean value from a triplet measurement. The error bars represented mean with standard errors of measurement (SEM).

## Results

### Strain-specific Progress of the VBNC State

To test if there were any differences in development of the VBNC state among the different strains, we analyzed the VBNC development curves for three *V. cholerae* strains, including the classical biotype strain O395 and the El Tor biotypes C6706 and N16961. The cells were cultured under the control condition (cells from the exponential-phase were incubated in ASW at 4°C with oxygen, see **Table 1**).

**FIGURE 1:**
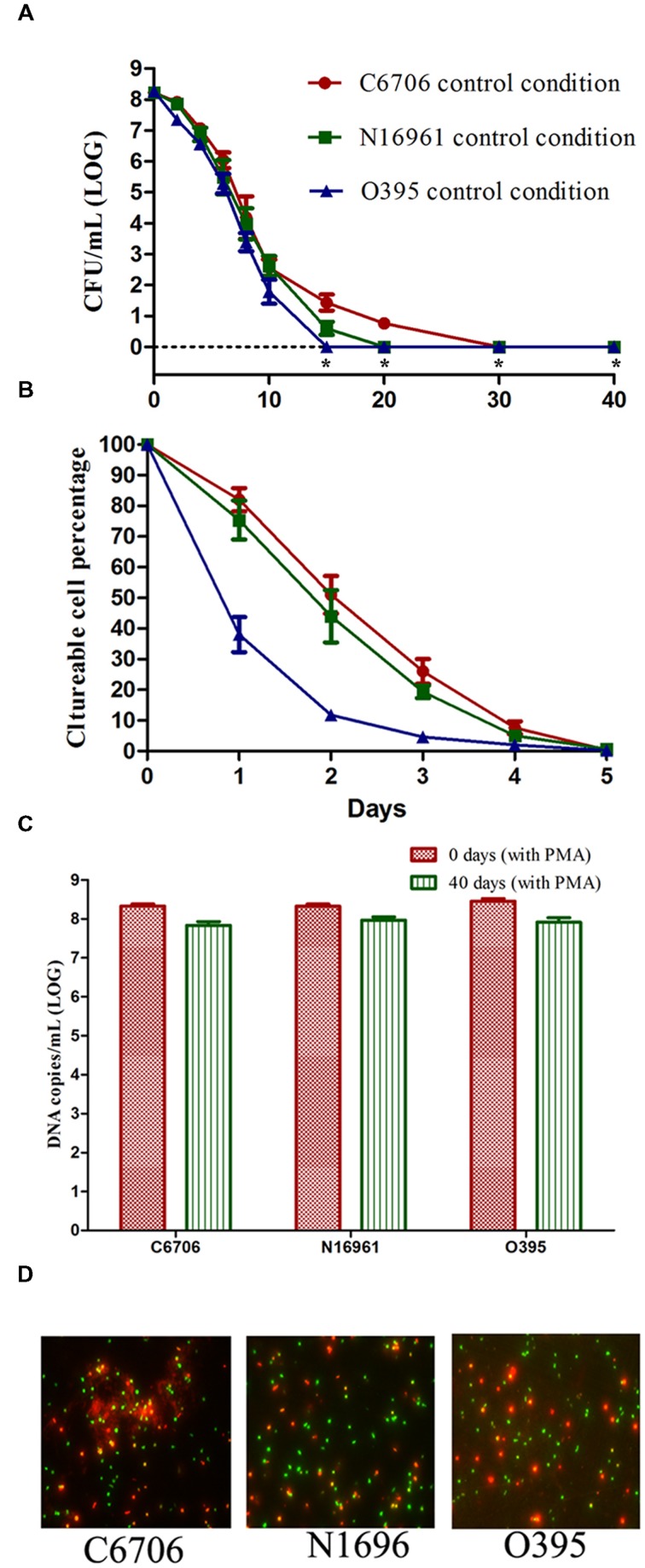
**Effect of strain on the development of the VBNC state. (A)** Progress curve of culturable cell counts until total loss of culturability. “*” indicates that the culturable cell count was less than 1 CFU/mL. **(B)** Progress curve of culturable cell counts during the first 5 days. The percentage of culturable cells was measured by the culturable cell counts at each time point/the culturable cell counts at the beginning. **(C)** DNA copies/mL from viable cells at days 0 and 40 (after PMA treatment). **(D)** Live/dead staining at day 40. The legends of part **(A–D)** are appropriate for both **Figures 2** and **3**. Error bars represent mean with SEM.

As shown in **Figure 1A**, the culturable cell counts of all three strains decreased gradually over the 40 days of incubation on TSA-SP agar with different progress curves. The time taken to reach 100% loss of culturability (LCT100) for O395, C6706, and N16961 were 15, 30, and 20 days, respectively. During the first 5 days of VBNC induction, the classical strain lost the ability to form colonies at a faster rate than the El Tor strains (**Figure 1B**). The LCT50 and LCT90 of the El Tor strains occurred both 1 day later than the classical strain. The progress curves of the El Tor strains were similar during the first 5 days. On day 40 after culture, the viable cells of all three strains were confirmed by PMA-qPCR and live/dead staining (**Figures 1C,D**). The mean viable cell counts, which were estimated from the DNA copies of viable cells, slightly decreased from 10^8.33-8.46^ to 10^7.84-7.97^ copies/ml, while no cultivable cells could be found.

These results suggested that the development of the VBNC state of *V. cholerae* might progress differently depending on the strain. The culturability of the O1 classical strain O395 decreased more quickly than that of the O1 El Tor strains C6706 and N16961. Even within the same biotype of El Tor strains, C6706 lost 100% culturability 10 days after N16961, although no significant differences were found between the progress curves of the two strains from the early stages of VBNC induction.

### Growth Phases Affect VBNC Progress

**FIGURE 2:**
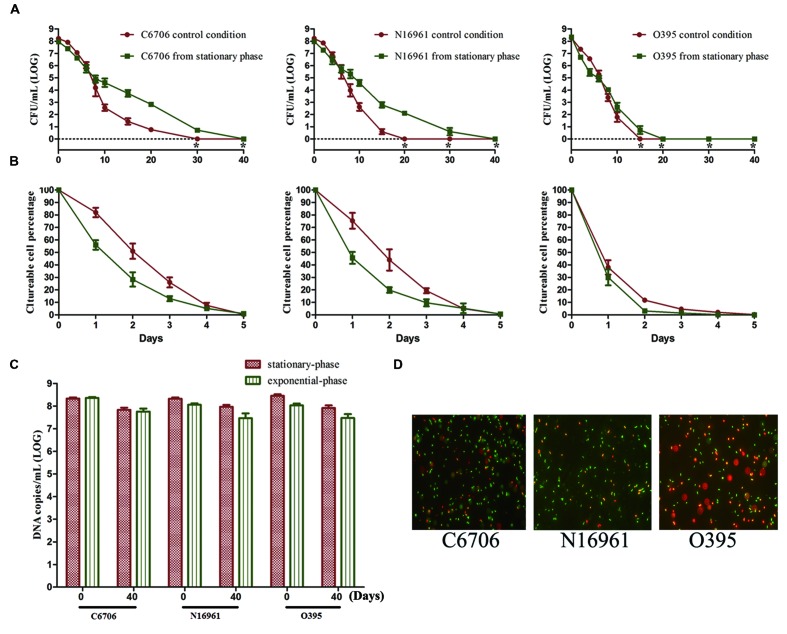
**Effect of growth phase on the development of the VBNC state.** Cells from the exponential-phase were incubated under the control conditions. **(A)** Progress curve of culturable cell counts until total loss of culturability. “*” indicates that the culturable cell count was less than 1 CFU/mL. **(B)** Progress curve of culturable cell counts during the first 5 days. The percentage of culturable cells was measured by the culturable cell counts at each time point/the culturable cell counts at the beginning. **(C)** DNA copies/mL from viable cells at days 0 and 40 (after PMA treatment). **(D)** Live/dead staining at day 40. Error bars represent mean with SEM.

To study the effect of growth phase on progress of the VBNC state, we compared the effect of stationary and exponential-phase cells when used as the starting cells in the VBNC experiment. All three test strains from the stationary-phase were found to maintain culturability for a longer time than cells from the exponential-phase (**Figure 2A**). The LCT100 for stationary-phase C6706 and N16961 cells was 40 days, while for exponential-phase C6706 and N16961 cells it was 30 and 20 days, respectively. During the VBNC incubation for these two strains, there were approximately 100-fold more culturable cell counts from stationary-phase cells than from exponential-phase cells from the 10th day of the incubation to the 20th day. Although, the growth phase showed a milder effect on VBNC development in the O395 strain, the LCT100 for stationary-phase cells was still 5 days later than exponential-phase cells.

However, in all three strains, most cells lost their culturability faster in the stationary-phase than in the exponential phase at the early stage of the induction (**Figure 2B**). The LCT50 and LCT90 for the El Tor strains and the LCT90 for O395 were all delayed when exponential phase cells were used. Together with the results of LCT100, as only a small proportion of cells could keep their culturability for a longer duration (**Figure 2A**), the heterogeneity of cells should be considered. The results of PMA-qPCR and live/dead stain showed that all the strains from both phases entered into VBNC state and no significant differences was found (**Figures 2C,D**).

### Oxygen Limitation Postponed VBNC Development

To evaluate the effect of oxygen on the development of the VBNC state, the three strains were incubated in ASW at 4°C in the presence of oxygen or in oxygen-limited conditions (**Table 1**).

**FIGURE 3:**
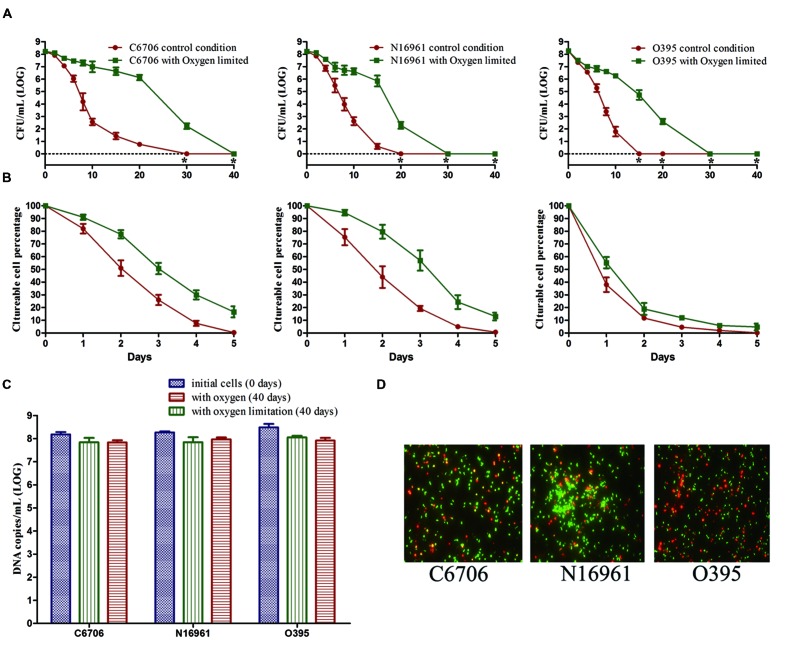
**Effect of oxygen on the development of the VBNC state.** The control conditions included aeration of the cells during the induction. **(A)** Progress curve of culturable cell counts until total loss of culturability. “*” indicates that the culturable cell count was less than 1 CFU/mL. **(B)** Progress curve of culturable cell counts during the first 5 days. The percentage of culturable cells was measured by the culturable cell counts at each time point/the culturable cell counts at the beginning. **(C)** DNA copies/mL from viable cells at days 0 and 40 (after PMA treatment). **(D)** Live/dead staining at day 40. Error bars represent mean with SEM.

During the first 20 days of the incubation, the mean culturable cell counts of strain C6706 slightly decreased from 10^8.22^ to 10^6.12^ CFU/mL with oxygen limitation while it decreased to 10^0.76^ CFU/mL in the presence of oxygen. The LCT50, LCT90 and LCT100 for the oxygen-limited condition were 1, 2, and 10 days later than the control (oxygen) condition, respectively (**Figures 3A,B**). The live cell counts determined by PMA-qPCR showed that cells in both conditions entered the VBNC state, and there were no significant differences between the two conditions after 40 days of incubation (**Figure 3C**). The VBNC state was confirmed by live/dead staining (**Figure 3D**).

The N16961 and O395 strains showed a similar progress under the oxygen and oxygen-limited conditions (**Figure 3**). These data suggested that oxygen limitation had little influence on the VBNC outcome, but could postpone the progress of VBNC development.

### Low Temperature is Necessary for VBNC Induction in ASW

The effects of the incubation temperatures for the induction of VBNC state were also compared by incubating the three strains in ASW at 4, 22, and 37°C (**Table 1**).

**FIGURE 4:**
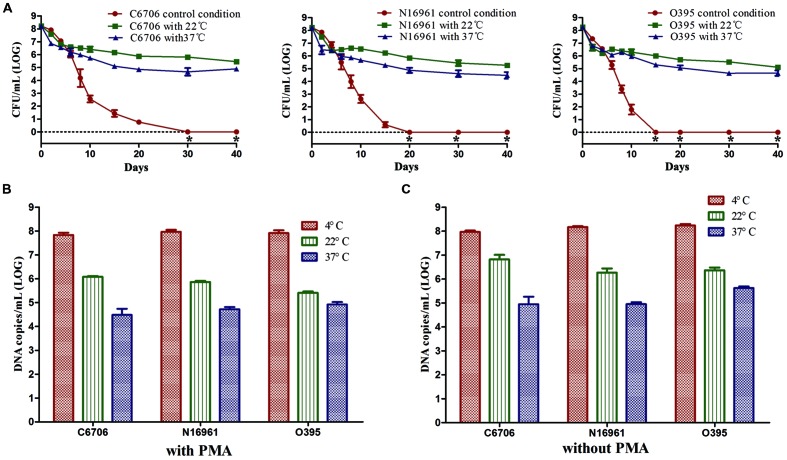
**Effect of temperature on the development of the VBNC state.** The control conditions mean the induction was performed at 4°C. **(A)** Progress curve of culturable cells until total loss of culturability. **(B)** DNA copies/mL from viable cells at 40 (with PMA). **(C)** DNA copies/mL from total cells at day 40 (without PMA). Error bars represent mean with SEM. “*” indicates that the culturable cell count was less than 1 CFU/mL.

We found that temperature was a crucial factor for the formation of the VBNC state. Compared with the control condition, the culturable cell counts of all three strains rapidly dropped from about 10^8^ CFU/mL to about 10^6^ or 10^5^ CFU/mL for 22 or 37°C, respectively (**Figure 4A**). Then, cells maintained culturability to at least 40 days at these two temperatures. PMA-qPCR results revealed that the live cell counts decreased to about 10^6^ copies/mL and 10^5^ copies/mL at 22 and 37°C, which were at the same level of the culturable cell counts after incubation for 40 days (**Figure 4B**). The total cell counts calculated in terms of total DNA copies by qPCR (without PMA) were significantly decreased at 22 and 37°C compared to 4°C, indicating that large numbers of cells decomposed at the two higher temperatures (**Figure 4C**).

These results suggested that a higher temperature might prevent VBNC development. At 22 or 37°C in ASW, most of the cells died rapidly and only about 0.1–1% of the cells could maintain their culturability for a longer time.

### Nutrient-rich Environment Leads to a Failure of VBNC Development

To test the effects of nutrient rich environment on VBNC development, the three strains were incubated at 4°C in ASW supplied with LB (ASW-LB, **Table 1**).

**FIGURE 5:**
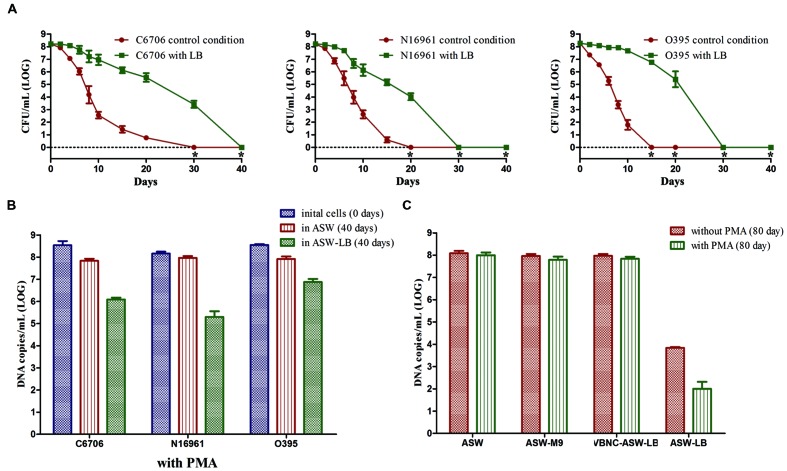
**Effect of nutrients on the development of VBNC state.** Control conditions mean the induction was performed in ASW. **(A)** Progress curve of cultivable cell count until total loss of culturability. **(B)** DNA copies/mL from viable cells at days 0 and 40 (with PMA). **(C)** DNA copies/mL of strain C6706 from viable cells and total cells at day 80. Error bars represent mean with SEM. “*” indicates that the culturable cell count was less than 1 CFU/mL.

There was a much slower decline in the culturable cell counts of strain C6706 in ASW-LB than in ASW. At day 30, when there were no culturable cells in the control condition (ASW at 4°C), 10^3.42^ CFU/mL of culturable cells were found in ASW-LB. No colonies were formed from ASW-LB after the 40 days incubation period (**Figure 5A**). After 40 days, PMA-qPCR showed that the DNA copies of viable cells dropped from about 10^8^ to 10^6^ copies/mL in ASW-LB, while no live cells could be found by live/dead stain. The other strains showed similar results (**Figures 5A,B**).

Cells in the VBNC state are supposed to maintain their viability for a longer time. To verify whether the cells cultured in ASW-LB had achieved the VBNC state after incubation for 40 days, LB broth was added to the control medium (VBNC-ASW-LB), and the cells were then incubated at 4°C for another 40 days. qPCR was then performed with and without PMA (PMA was added for the viable cell counts, and the qPCR without PMA was carried out to determine the total cell counts). The results showed that the cell counts in both ASW and VBNC-ASW-LB were similar, while the cell counts declined to about 10^4^ and 10^3^ copies/mL in ASW-LB condition, respectively. These results suggested that the VBNC cells can remain viable in ASW-LB; however, the “viable” cells calculated by the PMA-qPCR in ASW-LB condition were not VBNC cells but dead cells that had gradually decomposed (**Figure 5C**).

To test whether the presence of carbon and nitrogen sources could impede the VBNC phase, *V. cholerae* strains cultured in ASW-M9 medium were also determined. The strains achieved the VBNC state in this medium, and there were no significant differences in the viable cells compared with the control condition (in ASW) after 80 days of incubation (**Figure 5C**).

## Discussion

The VBNC state is the response of bacteria to rigorous environments such as low nutrition, low temperature, and unsuitable pH. In this study, we determined the effects of different culture conditions on the development of the VBNC state among toxigenic *V. cholerae* serogroup O1 strains.

Viable but non-culturable development progresses with loss of culturability and preservation of viability (Trevors, 2011). Therefore, viable cell counts and culturable cell counts were the main indicators to reflect the progress of VBNC development. The results of the viable cell counting implied whether or not the VBNC state would be induced. The LCT50, LCT90, and LCT100 could be used to indicate the speed of the development of VBNC state to determine which conditions induce or impede the development of VBNC.

Of the five induction factors studied, strain, growth phase, and oxygen level did not affect the outcome of the VBNC state in ASW at 4°C. When no culturable cells were found in 1 mL microcosms, 28.7–43.8% of the cells from the initial population maintaining viability. However, all the three factors influenced the speed of formation of non-culturable cells.

Several studies have reported differences in VBNC progress across different bacterial strains (Besnard et al., 2002; Pinto et al., 2011). We found differences in the VBNC progress varied not only within the same species but also within the same serotype, and the same biotype in *V. cholerae*. *V. cholerae* strain O395 exhibited a more rapid loss of culturability than the *V. cholerae* O1 El Tor strains. Within the same biotype, C6706 needed longer time to achieve total loss of culturability than N16961 in ASW at 4°C, although there were no significant differences in the LCT50 and LCT90 between the two strains. These findings suggested that the differences were likely related to the similarity of the genetic backgrounds of the *V. cholerae* strains.

Exponential-phase cells were found to enter the non-culturable state faster than stationary-phase cells. Similarly, previous studies of *Aeromonas hydrophila* and *E. coli* have also pointed out that the stationary-phase could delay the development of the VBNC state (Reissbrodt et al., 2002; Maalej et al., 2004). However, the LCT50 and LCT90 values in our study revealed that most of the cells lose their cultivability faster in the stationary phase. Furthermore, after no clones were formed in the exponential-phase, the stationary phase cells only produced <10 CFU/mL. As the initial cell number of 10^8^/mL, this proportion of cells cannot represent the total mass. Therefore, although we found that the growth phase may influence the progress of VBNC, there is insufficient data to confirm whether the stationary-phase causes delays in reaching the VBNC state in *V. cholerae*.

Not much is known about the effect of oxygen on the development of VBNC. To our knowledge, the only two studies investigating this have presented contradictory results. One study on *Campylobacter jejuni* reported that aeration could accelerate the development of the VBNC state (Rollins and Colwell, 1986), whereas in the other study, aeration was found to delay the development of VBNC for wine microorganisms (Millet and Lonvaud-Funel, 2000). Previous studies showed that the commercially available antioxidant Oxyrase helped resuscitate *Salmonella enterica* and *E. coli* from the VBNC state, although the influence of oxygen on VBNC development was not mentioned in the studies (Reissbrodt et al., 2002). In the present study, oxygen limitation obviously delayed the development of the VBNC state. The largest difference in the CFU between the presence and absence of oxygen reached nearly 10^6^-fold in all the three strains, suggesting that aerobic metabolism or oxygen injury might play a part in the mechanism of VBNC development.

The other two factors, temperature and starvation, have been most often used in previous studies to induce the VBNC state (Oliver, 2005; Trevors, 2011; Pinto et al., 2015). It is well-known that the VBNC state can be induced in many bacterial species by a combination of low temperature and starvation (Rollins and Colwell, 1986; Hoff, 1989; Oliver et al., 1991; Bovill and Mackey, 1997; Ghezzi and Steck, 1999; Boaretti et al., 2003; Fera et al., 2008). However, the role of these two factors have not been well-studied. Here, we found that the development of VBNC could be blocked simply by elevating the temperature or by adding LB to the culture medium. Increasing the temperature of the control condition in ASW may lead to a rapid decomposition of cells whose nutrients might be then used to maintain the culturability of the surviving cells. These results indicated that a low temperature is necessary to induce the VBNC state of *V. cholerae* in ASW. It is interesting that temperature alone may work as a “switch” to control the outcome of VBNC induction in ASW. It would be useful to find out the exact temperature that initiates the VBNC process. Comparative studies over a small range of the temperature might provide helpful clues to the genetic regulation behind the VBNC development.

Starvation was not necessary in order to attain the VBNC state for *V. cholerae*. VBNC development did not occur when the cells cultured in ASW at 4°C were supplied with LB; however, VBNC induction was not affected when cells were cultured in ASW with M9 at 4°C. Comparison of the ASW-LB and ASW-M9 results suggested there should be some differences between the two conditions that may have resulted in a failure of cells to enter the VBNC state. The different components of LB should be added to ASW-M9 individually to determine which component affects the cells and VBNC state, in order to obtain more clues into the development of VBNC.

The knowledge of the VBNC state is to date extremely limited. One reason for this is the complicacy of inducing conditions. Without the awareness of the role of each individual factor composing the inducing condition might result in the instability of outcomes. Understanding the role of each factor, including both those facilitating and those blocking the VBNC state, will assist our understanding of VBNC development. In this study we estimated the role of the growth phase, oxygen, temperature, and nutrient starvation in the development of the VBNC state in *V. cholerae*, providing useful data for and impetus for further studies into how the VBNC state is triggered and the regulatory mechanisms.

## Author Contributions

BW contributed to the study design, the experiment operation, data analysis, and paper writing. WL contributed to the study design, experiment operation, and data analysis. BK contributed to the study design, data analysis and paper writing.

## Conflict of Interest Statement

The authors declare that the research was conducted in the absence of any commercial or financial relationships that could be construed as a potential conflict of interest.

## Supplementary Material

DATA SHEET 1 **Culturable cell counts and viable cell counts**.
